# Canavanine-induced longevity in mice may require diets with greater than 15.7% protein

**DOI:** 10.1186/1743-7075-2-7

**Published:** 2005-02-25

**Authors:** Dan L Brown

**Affiliations:** 1 Animal Science Department, Cornell University, 320 Morrison Hall, Ithaca, NY 14853 USA

## Abstract

**Background:**

Dietary administration of 1% canavanine had been shown to improve survival in female BALB/c mice consuming diets containing 23.4% protein (dry matter basis).

**Methods:**

In order to determine if this effect also obtains at more moderate dietary protein concentrations, 30 female BALB/c mice were fed a basal diet with 14% protein (15.7% dry matter basis) and another 30 were fed the same diet plus 1% canavanine.

**Results:**

Neither mean (Control 873.2 d, Canavanine 870.0 d; SEM = 34.2 d; P = 0.949 from ANOVA) nor median (Control 902 d, Canavanine 884.5 d; P = 0.9058 from Mann-Whitney) lifespans differed between groups.

Although mean antinuclear antibody (ANA) titers did not differ between control and canavanine-treated mice at 833 days of age (19.84 vs 20.39 respectively; SEM = 2.64; P = 0.889 from ANOVA), one canavanine-treated mouse displayed an outlying ANA value of 50 (next lower value = 30) denoting possible early sign of incipient autoimmune disease in that individual.

**Conclusion:**

There may be an interaction between dietary protein level and canavanine with respect to lifespan in mice.

## Background

**Figure 1:**
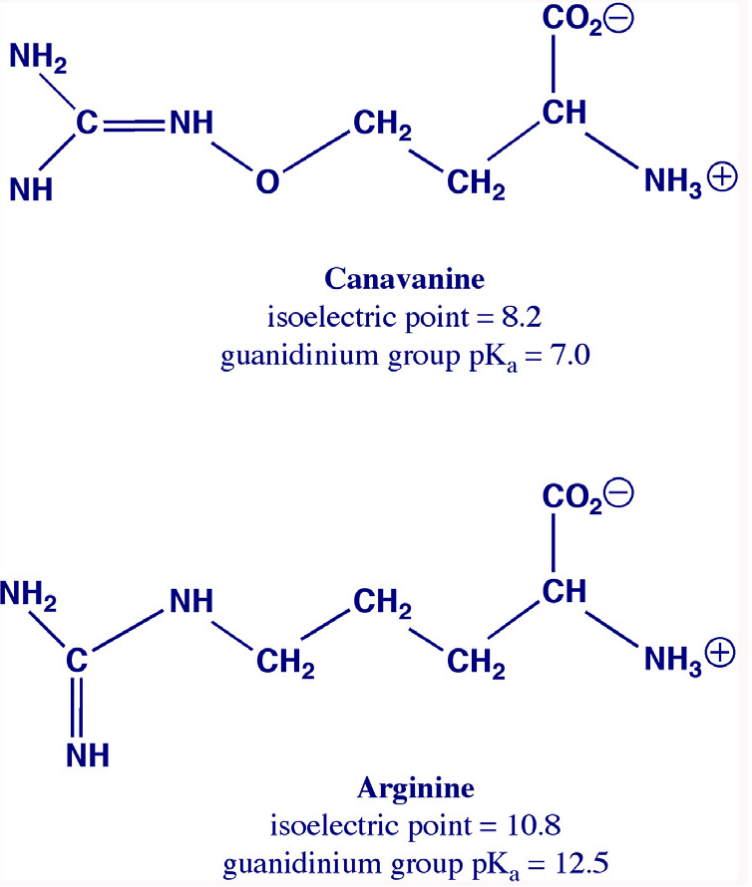
Chemical structure of canavanine and arginine

L-canavanine is a common non-protein amino acid found naturally in alfalfa sprouts, broad beans, jack beans, and a number of other legume foods and animal feed ingredients [1] at up to 2.4% of food dry matter. This analog of arginine (Figure 1.) can also block NO synthesis [2-5], interfere with normal ammonia disposal [6,7], charge tRNAarg, cause the synthesis of canavanyl proteins [8], as well as prevent normal reproduction in arthropods [9] and rodents [10].

Canavanine has also been reported to induce a condition that mimics systemic lupus erythematosus (SLE) in primates [11,12], to increase antibodies to nuclear components and promote SLE-like lesions in auto immune-susceptible (e.g., (NZB X NZW)F1) mice [13].

In our previous study [14], eighteen female BALB/c mice were fed a 23.4% protein diet containing 1.56% L-canavanine sulfate (equivalent to 1% L-canavanine base) and eighteen control mice received control diet (23.4% protein) from 84–477 days of age. More canavanine-fed mice (16 of 18) survived to age 477 days than those fed the basal diet (9 of 18) (X ^2 ^with Yates correction = 24.8).

The death rate and median life span of control mice were consistent with previously reported survival studies of female BALB-C mice fed high protein diets [15]. Necropsy and histopathology from both experimental animals and co-housed cages of contemporary sentinel mice performed by the University of California Comparative Pathology Laboratory (Davis, Ca.) did not detect any significant gross or histopathological lesions, parasites or pathogenic bacteria, viruses or mycoplasma. Serology results were negative as well. There was no evidence of infectious disease and no evidence of the lung tumors often found in older BALB-C mice [15]. One sentinel, but no experimental animals, showed evidence of a lymphosarcoma.

Kristal and Yu [16] have suggested that aging "...results in deleterious changes in cellular, intercellular, tissue and organismic functions."

Apparently, canavanine postponed the type of physiological decline that results in earlier death due to the inability of these aging mice to maintain homeostasis in the face of undetectable disease or some other stress.

These were serendipitous findings during a project designed to explore the mechanism of diet-induced autoimmunity. Ironically, no positive ANAs or anti SSDNA or anti dsDNA titers were detected in these mice as a result of canavanine treatment.

Previous reports of life span extension by restricting the intake of energy [17], protein [18], tryptophan [19] and methionine [20] have been accompanied by severe reductions in growth and eventual body size relative to controls. The severe stunting usually associated with amino acid deficiency and extended life span was not observed in the BALBc mice [14].

Our earlier results did leave unanswered the question of whether canavanine was extending life or merely offsetting the life-shortening effects of a very high protein diet. Since the objectives of the original investigation were unrelated to lifespan determination and required the harvest of body tissues, full lifespan data were not available.

The purpose of the experiment reported here was to test the hypothesis that canavanine will reduce middle age mortality and extend the lifespan of female BALB/c mice consuming a diet containing moderate concentrations of protein (15.7% DMB).

## Methods

Five female 100-day-old 20-gram BALB/cAnHsd mice (first generation offspring of mice obtained from Harlan Sprague-Dawley) were assigned to each of twelve cages and six cages were randomly assigned to each of two treatments: Control = 25 g of ground Agway 1000 lab animal feed or Canavanine = 25 g of a mixture containing 1.56% canavanine sulfate (prepared by the method of Rosenthal [21]) and 98.44% ground Agway 1000 lab animal feed resulting in a diet containing 1% L-canavanine base (Table 1). All animals were weighed monthly for the first 28 months of age and cause of death determined by symptoms and necropsy. As animals died, feed offerings were reduced, but 5 grams per head ratio was maintained.

**Table 1:** Experimental Diets

**Nutrient**	**Basal (Agway Prolab 1000)**	**Basal Diet + canavanine**
Dry Master %	89.88	89.78
All others on DMBasis		
NX6.25 %	15.70	17.84
Lipids %	7.05	6.94
Ash %	6.54	6.44
Gross Energy kcal/g	4.5	4.5
Estimated Metabolizable Energy kcal/	3.3	3.3
Calcium %	0.90	0.89
Phosphorus %	0.8	0.79
Arginine %	0.81	0.80
Canavanine %	-----	1.05

At 833 days of age, blood samples were taken from the supraorbital sinus of the 37 surviving mice (19 controls and 18 canavanine-fed) and analyzed for antinuclear antibodies (ANA) by the methods of Naiki, et al [22].

Variations in lifespan and ANA values were evaluated by analyses of variance and median values were contrasted by the Mann Whitney procedure [23].

## Results

**Figure 2:**
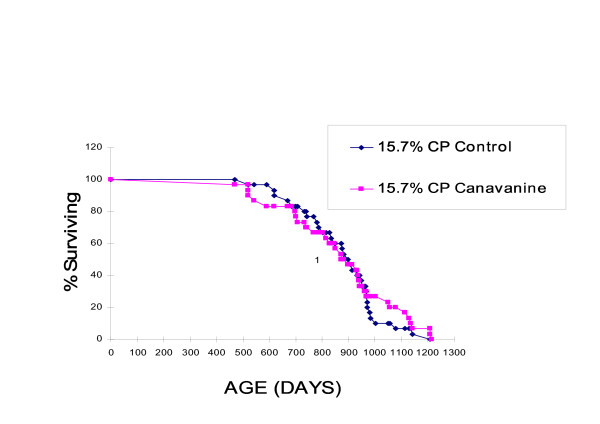
Survival of mice fed 15.7% dietary protein

Neither mean (870.0 vs 873.2, SEM = 34.2) nor median (885 vs 902, W = 923.5) lifespan differed significantly between mice fed canavanine and those fed the same basal diet without canavanine, respectively. (Figure 2).

**Figure 3:**
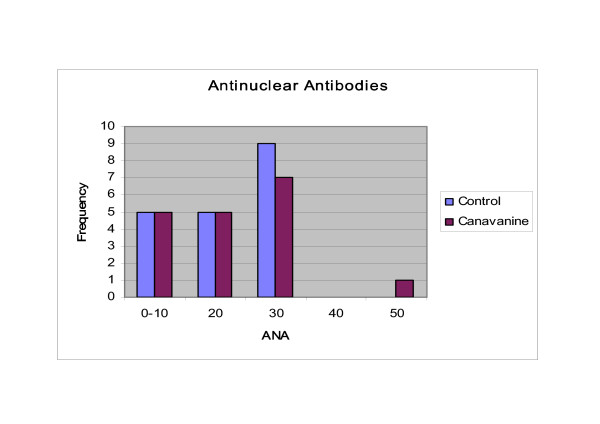
Antinuclear Antibodies

Although mean (20.39 vs 19.84) indices of ANA titers at 833 days of age were also similar for canavanine and control treatments, one canavanine-fed mouse displayed an unusually high titer (10X SEM above mean). (Figure 3).

About half of the mice (48.3%) displayed mammary tumors, pulmonary adenocarcinomas or both at the time of death (17 canavanine mice and 12 controls; but the difference was not significant by Chi-square analysis, [23]) (Table 2). No infectious diseases were detected in either experimental or sentinel mice.

**Table 2:** Probable cause of death

	**Mammary Tumor**	**Pulmonary Adenocarcinoma**	**Both Types of Tumor**	**Total With Any Tumor**	**Other**	**Undetermined**
Canavanine	9	7	1	17	2	11
Control	6	4	2	12	3	15
X^2^(Yates)	0.44	0.37	0.56	0.35	0.65	0.43
P	>0.50	>0.50	>0.30	>0.50	>0.30	>0.50

## Discussion

The median longevity of control mice observed in this experiment (in which a 15.7% protein was fed) considerably exceeds that of the previous trial in which the basal diet contained 23.4% protein (902 d vs 449 d). Storer [24] fed BALB/c mice a diet of protein content between those two extremes (17%, but probably closer to 19% on a dry matter basis) and observed mean longevity for females of 575 d, a result falling between our two observations. That decreasing protein concentrations should extend rodent lifespan is not a unique finding, the phenomenon is well known. The apparent corrective effect of canavanine at high protein concentrations and lack of effect at more moderate concentrations does offer a new dimension to dietary modulation of longevity that bears further study.

A number of populations consume protein in amounts that far exceed known nutritional requirements (humans in some Western countries, alfalfa-fed horses in some Western States and pet dogs in most of the Northern Hemisphere). The impact of these protein intakes on human and domestic animal longevity and the possible corrective effects of non-protein amino acids on those effects should interest a variety of medical, veterinary and nutritional workers.

For the second time, we have failed to induce significant autoimmune disease signs with dietary canavanine in BALB/c mice. Since this can be done in autoimmune susceptible strains and hybrids and in the "normal" (DBA/2) strain of mice [13], genetic differences may play a role in an animal's response to this secondary plant compound.

These preliminary experiments were not contemporary comparisons, nor were they conducted with both sexes, with the same source of BALB/c mice, the same basal diets or at the same locations. Known autoimmune-susceptible lines were not challenged in those trials. Although the data from those trials did indicate a canavanine-protein concentration interaction, we propose a contemporary, comprehensive, integrated experiment including both autoimmune susceptible and more normal populations. The proposed work will be more conclusive and provide an opportunity to examine the generality of the canavanine longevity effect, suggest mechanisms for its occurrence and find out if positive effects on oxidation event-dependent aging might be offset by autoimmunity in susceptible individuals. Examination of such indices as tissue and urine nitrate/nitrite, 8-OH deoxyguanidine, canavanyl residues in specific and total proteins would all contribute to a better understanding of mechanisms of longevity modulation.

## Conclusion

This and a previous study suggest that an interaction between dietary protein concentration and canavanine effects on longevity may exist in BALB/c mice. Contemporary trials that vary both protein and canavanine concentrations are needed to test this possibility more conclusively.

## Competing interests

The author(s) declare that they have no competing interests.

## References

[B1] Bell EA (1978). Non-protein amino acids in leguminosae. Advances in legume systematics.

[B2] Hrabak A, Bajor T, Temesi A (1994). Comparison of substrate and inhibitor specificity of arginase and nitric oxide (NO) synthase for arginine analogues and related compounds in murine and rat macrophages. Biochem Biophys Res Commun.

[B3] Krishna G (1994). Inhibition of brain nitric oxide synthase expressed on CHO cells by canavanine. NIH Project number Z01HL04421-94.

[B4] Umans JG, Samsel RW (1992). L-Canavanine selectively augments contraction in aortas from endotoxemic rats. European Journal of Pharmacology.

[B5] Dokita S, Smith SD, Nishimoto T, Wheeler MA, Weiss RM (1994). Involvement of nitric oxide and cyclic GMP in rabbit urethral relaxation. Eur J Pharmarol.

[B6] Thomas DA, Rosenthal GA (1987). Toxicity and pharmacokinetics of the nonprotein amino acid L-canavanine in the rat. Toxicology and Applied Pharmacology.

[B7] Thomas DA, Rosenthal GA (1987). Metabolism of L-[guanidinooxy-14]canavanine in the rat. Toxicology and Applied Pharmacology.

[B8] Crine P, Lemieux E (1982). Incorporation of canavanine into rat pars intermedia proteins inhibits the maturation of pro-opiomelanocortin, the common precursor to adrenocorticotropin and beta-lipotropin. J Biol Chem.

[B9] Rosenthal GA, Reighart JM, Hoffman JA (1989). L-canavanine incorporation into vitellogenin and macromolecular conformation. J Biol Chem.

[B10] Brown DL (1994). Reproductive toxicity and increased longevity in mice fed L-canavanine. J Anim Sci.

[B11] Malinow MR, Bardana EJ, Pirofsky B, Craig S, McLaughlin P (1982). SLE-like syndrome in monkeys fed alfalfa sprouts: role of a non-protein amino acid. Science.

[B12] Montanaro A, Bardana EJ (1991). Dietary amino acid-induced systemic lupus erythematosus. Rheumatic Disease Clinics of North America.

[B13] Prete PE (1985). Effects of L-canavanine on immune function in normal and autoimmune mice: disordered B-cell function by a dietary amino acid in the immunoregulation of autoimmune disease. Can J Physiol Pharmacol.

[B14] Brown DL, Naiki M, Gershwin ME (2000). Does L- canavanine ingestion induce murine SLE? Paradoxical effects on survival of BALB/c mice. J Nutr Immun.

[B15] Roscoe B, Jackson Memorial Laboratory Staff (1966). Biology of the Laboratory Mouse.

[B16] Kristal BS, Yu BP, Yu BP (1994). Modulation of Aging Processes by Dietary Restriction.

[B17] McCay CM (1935). The effect of retarded growth upon the length of life span and upon the ultimate body size. J Nutr.

[B18] Leto S, Kokkonen GC, Barrows CH (1976). Dietary protein, life-span and biochemical variables in female mice. J Gerontol.

[B19] Ooka H, Segall PE, Timiras PS (1988). Histology and survival in age-delayed low-tryptophan-fed rats. Mech Aging Dev.

[B20] Orentreich N, Matias JR, DeFelice A, Zimmerman JA (1993). Low methionine ingestion by rats extends life span. J Nutr.

[B21] Rosenthal GA (1977). Preparation and colorimetric analysis of L-canavanine. Anal Biochem.

[B22] Naiki M, Yasuyuki I, Yuhsuke K, Osawa T (1988). Effect of an autoreactive T cell clone from (NZB X NZW) F1 mice on the production of anti-DNA antibodies in vivo and in vitro. Immunology Letters.

[B23] Snedecor GW, Cochran WG (1980). Statistical methods.

[B24] Storer JB (1966). Longevity and gross pathology at death in 22 inbred mouse strains. J Gerontol.

